# Lisa K. Traditi, AHIP, Medical Library Association President, 2020–2021

**DOI:** 10.5195/jmla.2020.1100

**Published:** 2020-10-01

**Authors:** Brittany R. Heer, Ruby L. Nugent

**Affiliations:** 1 bheer@butler.edu, Health Sciences Librarian, Butler University Libraries, Butler University, Indianapolis, Indiana, IN; 2 ruby.nugent@unlv.edu, Dental Medicine Liaison Librarian, Health Sciences Library, University of Nevada, Las Vegas, NV

## Abstract

In this profile, Lisa K. Traditi, MLS, AHIP, Medical Library Association president, 2020–2021, is described as an individual with a bright personality, rich professional experiences, and a natural ability to lead. She is a respected mentor in the medical librarianship field, especially in the realm of evidence-based medicine instruction and education. Traditi has spent the past twenty-six years at the Strauss Health Sciences Library at the University of Colorado Anschutz Medical Campus.

Ebullient. Enthusiastic. Hysterical. Authentic. Collaborative. Vivacious. Amazing. Generous. Compassionate. Supportive. Effortless style. A natural leader.

When asked to describe her, these are the adjectives and phrases her friends and colleagues chose for Lisa K. Traditi, MLS, AHIP. How can one individual encompass all of these qualities?

Much as her love of camping has taken her on scenic tours of the Southwest, Lisa's travels through librarianship have also been an adventure with forks in the road, beautiful and unexpected byways, and a little weather along the way. Let us roll up our sleeping bags and pack our campers to get to know Lisa and follow her journey to the Medical Library Association (MLA) presidency.

## EARLY CAREER IN THE DESERT

Like many others who find themselves in librarianship, Lisa did not initially set out to become a librarian; her dream was to become a journalist and write novels. After receiving her bachelor of arts degree in English and creative writing from Missouri State University, she dabbled in the publishing world in Phoenix by lending her talents to the now-defunct magazine, *Arizona Living.* She was the “jill of all trades” at the magazine: selling advertising, managing the circulation database, answering the phones, and writing short pieces. “The Great American Novel” it was not, but it was a foot in the door toward the journalism career she was working on.

**Figure:**
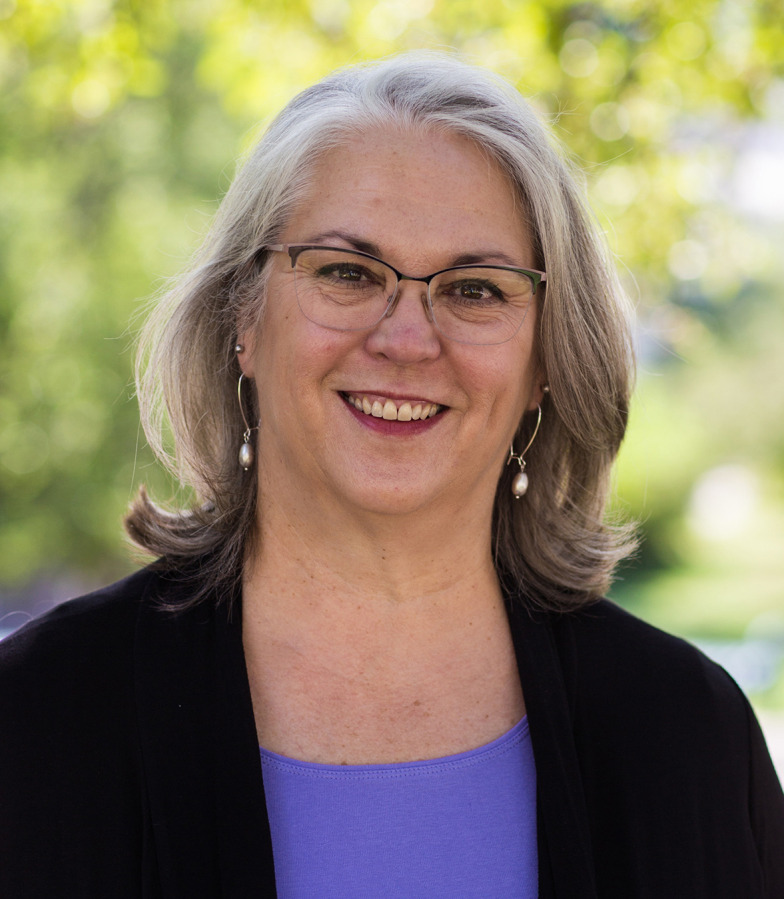


Her career in the publishing industry did not last long as she got an unexpected nudge toward the library world. Lisa's sorority, Alpha Delta Pi, was seeking graduate resident counselors to represent the chapter at the University of Arizona. Lisa could not resist such an opportunity, so she applied and was accepted with a full ride scholarship. Getting accepted was half the battle as she still was not sure what to study. Seeking guidance, Lisa presented herself to a librarian at the Phoenix Public Library, who suggested Lisa consider librarianship. She was intrigued and decided that she would pursue her master's in library science.

After graduating in 1984, Lisa immediately put her degree to use as a part-time reference librarian at the Scottsdale Public Library and then as the solo librarian at Garrett AiResearch in Tucson. But her life took another sudden turn when her then fiancé, now husband, accepted a position in Sioux City, Iowa. She quickly found a reference librarian role at Morningside College. Within a year, they transferred yet again, this time to Denver, Colorado, where her career and impact on the library community truly began.

## MEDICAL LIBRARIANSHIP IN THE MOUNTAINS

Lisa began working at the University of Denver as the librarian for the Social Systems Research and Evaluation (SSRE) Institute. However, within just six months, the university notified the program that it would be shut down. Never one to be discouraged, Lisa applied her unique qualifications of expert searching in online databases and found a hospital librarian position at Aurora Presbyterian Hospital (APH). In addition to managing the small physical collection, Lisa worked with medical residents and attended morning reports, grand rounds, and board meetings. She recalled this time as “terrifying and challenging and meaningful work, to be answering questions that had a direct impact on patient care.”

Lisa's “other duties as assigned” at APH included being the audiovisuals (AV) maven: troubleshooting the technical issues for conferences, grand rounds, and distance educational programs that used satellite systems. One day as Lisa was cursing the slide projector for yet another failure, colleague, mentor, and friend Karen K. Wells let her know that Wells's old position as library director at St. Luke's Hospital in Denver would be available soon. She recalled Lisa as “a bright star that was interested in so many things” and “ever eager to take on new challenges.” Lisa excitedly applied and transferred to St. Luke's. It was during her time at St. Luke's that Lisa would be exposed to evidence-based medicine (EBM) for the first time, which blossomed into her career-long passion as an EBM instructor and educator.

A few more mergers and libraries later, Lisa found her way to managing a small team of five at Swedish Medical Center Library. Building upon her previous experiences, she was tapped to be a leadership and management trainer for the hospital. Lisa noted how she learned “in-depth training and experience as a group facilitator and teacher,” much of which she continues to use to this day.

## STRAUSS HEALTH SCIENCES LIBRARY

The 1990s saw many hospital mergers, and Swedish Medical Center was no exception. Sadly, Lisa and her team were ultimately laid off, once again pushing Lisa to search for new opportunities. Within a few months, she accepted a position as head of education at Denison Memorial Library (now Strauss Health Sciences Library) at the University of Colorado (CU) Health Sciences Center (now University of Colorado Anschutz Medical Campus). At Denison, she oversaw the early growth of her department with grace and flexibility.

Rick B. Forsman, FMLA, director of Denison Memorial Library until 2008, described Lisa as:

[S]elf-directed, goal focused, so concerned about other people, always upbeat, collaborative, find[s] a way to work well with almost any personality type or perspective, and gets people to agree on how to move forward. She believes that any problem should have a solution and if a group is having trouble finding an answer, she immediately begins asking questions that might lead to new approaches in problem solving. She doesn't give up but tries new avenues.

## ROCKY MOUNTAIN EVIDENCE-BASED HEALTH CARE WORKSHOP

Lisa's tenacity and collaborative spirit would prove invaluable when she began serving as the librarian coordinator for the Rocky Mountain Evidence-Based Health Care (RMEBHC) Workshop. Through her work with the RMEBHC Workshop, she would have the pleasure of working with EBM luminaries such as David Sackett, Scott Richardson, Lisa Bero, Iain Chalmers, Alejandro Jadad, and Martha Gerrity, not to mention the many colleagues and friends she met and impacted along the way.

Former librarian tutors of the RMEBHC consistently underscored Lisa's organizational skills and her ability to bring together subject specialists alongside librarians. Stephanie Fulton, AHIP, Medical Sciences Library director and Texas A&M Libraries associate dean, remembered that participants “saw Lisa as a respected member of the organizing team and also as a gifted instructor. She [was] always willing to ask questions [and] find out information if she [did] not know something.” Margaret Moylan Bandy, AHIP, FMLA, colleague and mentor during Lisa's hospital librarianship days and RMEBHC tutor from 2002 to 2004, noted that even though “it was hard work and challenging, Lisa made it fun and professionally rewarding. Because of Lisa, I think the expertise that librarians can contribute to the practice of EBHC was quickly recognized by the participants, many of whom had never worked closely with librarians before the workshops.”

## SUPPORTING CLINICAL CARE: AN INSTITUTE IN EVIDENCE-BASED PRACTICE FOR MEDICAL LIBRARIANS

In 2014, Lisa began coordinating an EBM workshop at the CU Strauss Health Sciences Library. Originated by the Dartmouth University Biomedical Libraries, the “Supporting Clinical Care: An Institute in Evidence-Based Practice” is an intense, immersive workshop for medical librarians that spans five days and continues to be sought out by librarians from all over the world. Participants are broken up into small groups and led by expert medical librarian tutors who work closely with attendees to both understand and practice evidence-based research in preparation for providing similar instruction to their constituencies.

Amy Blevins, MALS, associate director for public services at the Ruth Lilly Medical Library at Indiana University, who was a participant and later a tutor for the program, stated that “Lisa has been well-regarded for her skills in teaching EBM for many years. The work she has done with Supporting Clinical Care: An Institute in Evidence-based Practice for Medical Librarians has inspired many librarians.”

Lynne Fox, AMLS, MA, retired colleague at CU's Strauss Health Sciences Library, highlighted both Lisa's and the library's successes “in preparing health professionals, students and librarians for evidence based practice [by improving] health care and [raising] the profile of medical librarians. That's a direct result of wanting to share her unique talents and those of her colleagues to lift up those around her.”

## LEADERSHIP THROUGH ROCKY TERRAIN

While Lisa's impact and influences in the EBM arena were, are, and continue to be far and wide, throughout it all, she steadfastly led her team at Strauss Health Sciences Library with compassion and understanding through departmental reorganizations and even a physical library move. Forsman reflected:

We had budget cuts, personnel crises, an exhausting move to a new campus, but through every damn thing she maintained her equanimity, did more than her part, and boosted the morale of other staff. She always looked for a bright side and helped others do the same.

Gerald (Jerry) J. Perry, AHIP, FMLA, former director of CU's Strauss Health Sciences Library and current associate dean for health sciences and for strategic planning at University of Arizona, fondly recalled that:

[Lisa's] humor, and her deeply compassionate approach to appreciating her colleagues, were grounding and brought perspective to even the most anxious of moments. She reminded me always of what was most important—the people and relationships in our lives—and how while so much may come and go, the quality of one's relationships [was] foundational and critical to remembering the “why” of what we were doing, and especially the “for whom.” An amazing teacher and learner, Lisa also reminded us of the many teachable moments we could embrace over the course of a day. She was particularly adept at doing this during our leadership meetings, where with her emotional intelligence and ability to read a room she could steer a conversation to consider possibilities when others were focusing on crisis, me included!

Melissa De Santis, MLIS, AHIP, director of the Strauss Health Sciences Library, emphasized Lisa's strategic and thoughtful approach to a restructuring of departments and personnel in 2010:

This was a huge culture shift for the library. As you might imagine, not everyone was happy about this reorganization. Lisa approached this challenge like everything else—with a positive attitude and an open mind. She got the new department talking about what they saw as the department's strengths, weakness, opportunities and threats. She took that feedback and helped the group create shared goals. She asked a lot of questions, and she listened a lot. She was empathic to staff that were unhappy, but she also held people accountable and did not allow unhappiness to manifest as bad behavior. She knew it was not going to be a quick fix, and it did take time, but the department is now highly successful and functioning very well.

In 2017, Lisa became the deputy director of Strauss Health Sciences Library. Fulton observed that “Lisa was very successful in her previous position and also comfortable. She knew what needed to be done for the job she had. [Taking] on additional responsibilities and challenges was not something she necessarily had to do but decided she would get out of her comfort zone and…her library has benefited.” Although she rarely does literature searches anymore, Lisa loves the challenges that this new phase of her career affords her.

## PUTTING DOWN STAKES IN PROFESSIONAL ASSOCIATIONS

In addition to her extensive contributions to her home libraries throughout her career, Lisa also lends her knowledge and talents to many professional associations. This is evident in Lisa's commitment to service to the profession, where colleagues recognize her ability to be enthusiastic and energetic in the various roles she has taken on. Fox noted that “Lisa Traditi has spent her career looking for ways to say yes. This has opened up tremendous opportunities for her and her library colleagues.”

### Colorado Council of Medical Libraries

Sara Katsh, MA, AHIP, library manager of the Association of periOperative Registered Nurses (AORN) from 1972–2014, remembered how Lisa “burst [onto] the medical library scene in 1987” through her work in the Colorado Council of Medical Librarians (CCML). Lisa quickly volunteered to serve on committees and take on leadership roles. Bandy reflected that Lisa's talents were quickly “recognized by her colleagues,” especially when she was president-elect in 1989 and president in 1990. CCML colleagues became both mentors and friends, and encouraged Lisa to continue to strengthen her skills and apply her knowledge. Lisa was honored by CCML in 2005 with the Marla Graber Award for Excellence and Achievement in Health Sciences Librarianship.

### Midcontinental Chapter of the Medical Library Association

Lisa expanded her professional network and became actively involved with the Midcontinental Chapter of the Medical Library Association (MCMLA), where she became more visible as a collaborator and leader that the membership both recognized and appreciated. Bandy reminisced on her time working with Lisa in MCMLA: “My memory is that when Lisa was involved, working on a project was more fun than it would have been without her.”

Lisa was elected chapter chair twice, in 2001 and in 2016. In 2004, she was awarded the chapter's Bernice M. Hetzner Award for Excellence in Academic Health Science Librarianship. In 2014, Denver hosted a Quint Chapter Meeting, which was held by five MLA regional medical library chapters. Lisa served as cochair of and MCMLA liaison to the Quint Chapter Meeting Steering Committee. Lisa elevated her presence in medical librarianship organizations to new heights with this feat that required organization, commitment to quality, and exceptional leadership.

### Medical Library Association

These significant achievements did not go unnoticed by those around her. She was encouraged by colleagues and mentors to use her “voice as an influence for good” in her local and regional chapter as well as a representative in MLA at large. Lisa took that to heart and spent much of her time in the organization volunteering to serve in several capacities, including participating in meeting planning committees and as a member of various task forces.

One of the most impactful roles Lisa took on was her time spent as an elected member of the Board of Directors. Those around her observed and welcomed her careful approach to addressing difficult issues and engaging in respectful exchanges among a diverse and dynamic group of individuals. Blevins explained that “Lisa has this amazing ability to encourage dialog and de-escalate tension.”

Lisa became known for her characteristic upbeat but goal-focused approach to collaboration. Her ability to bring colleagues together with deliberate questioning and gentle direction toward paths of consensus has made her stand out as a team member and leader. Teresa L. Knott, MLS, MPA, AHIP, FMLA, interim dean of libraries and university librarian Virginia Commonwealth University, highlighted Lisa as:

the consummate professional. She is knowledgeable about librarianship and advances in our field. She is innovative and a committed lifelong learner who is willing to transform her practice of librarianship based on evolving evidence. She is an outstanding and gracious educator who is genuinely interested in those fortunate enough to be sitting in a classroom with her leading the way. As a leader, Lisa is gracious, inclusive, and thoughtful.

## BLAZING HER TRAIL: THE MEDICAL LIBRARY ASSOCIATION PRESIDENCY

As she set her boots down upon the trail to her MLA presidency, Lisa acknowledged that this year will look quite different due to the COVID-19 global pandemic. Instead of her inaugural address being given in a large meeting room in Portland, Oregon, she presented via Webex from her home in Aurora, Colorado. While she misses the immediate feedback that comes with MLA annual meetings, she is thrilled that so many more members were able to attend virtually than have attended in-person annual meetings in the past. Once again, Lisa looks to the positive aspects of any challenge that may cross her path.

Lisa's goals for her term as president are to improve transparency and communication. She recognizes that “the Board of Directors and MLA presidents have always strived to be clear and communicate often.” However, after years of being deeply involved with the association, it is easy to forget what it is “like not to know about all the inner workings of MLA.” She intends to engage and connect virtually throughout the year via virtual forums and conversations with members and stakeholders. Lisa underscores that “the more we communicate with each other, the more we can stay connected and improve how MLA can provide value and assistance to our community.”

To achieve these goals, Lisa leans on appreciative inquiry and servant leadership, which she learned from Perry. Lisa explains:

I think my job is to move things out of the way so that those who report to me can get things done. I also strongly believe that leadership can happen at any level of an organization. Leadership is not about a title or position, although those with the title or position should be using their influence to lead. Some of the best leaders I've known are individual contributors, who work hard and inspire others to do better. Finally, the best leaders are always learning and working to grow their skills and mentor others into leadership roles.

While MLA needs leaders like Lisa to inspire others, she emphasizes the importance of MLA members' involvement. Lisa's advice to members is:

Say yes to opportunities, then show up and do the work asked of you with integrity. Volunteer for things you're interested in doing. If you're shy, ask someone you know to sponsor you and put your name up for something you want to do. MLA is run by us, the members, it's not a corporate entity, so if we want something to be better or different, we all have to work to make that happen. At the same time, be patient and extend grace to yourself and others who are involved in the association. We're all doing the best we can while still working full time jobs. Things may not move as quickly as we'd like, but as long as we can keep things moving in the right direction, we should count that as a win.

Although Lisa acknowledges volunteering her time has been a large part of her success as a librarian and leader, she also recognizes the dangers of overcommitting. She reflects on guidance given by Graber, her previous supervisor, about being respectful of others' time and not overcommitting. Lisa has never forgotten Graber's advice that “it's important to only accept those volunteer jobs you can do well and with integrity.”

## REFLECTING ON ROADS TRAVELLED AND LOOKING TOWARD THE HORIZON

When asked to reflect on their fondest memories of Lisa, there was a clear theme among those who know her best. From the very beginning, Lisa has exhibited her natural humor, personability, and overall positive attitude in everything she does. She strives to be present and engaged with her peers and has an innate ability to not only hear different perspectives, but to also include others in the conversation. Catherine Reiter, former collection management librarian at CU's Strauss Health Sciences Library, who first met Lisa through CCML meetings, described her as “incredibly funny, friendly, and cheerful…with a great interest in people.”

Lisa's bright personality, rich experience, and natural ability to lead has also established her as a well-known and well-respected mentor in the medical librarianship field. Many have benefited from Lisa's genuinely human approach to counseling not only the professional struggles of a mentee, but the personal ones as well. She provides honest and helpful feedback for those seeking it, both formally or informally, and often over a meal. Coauthor Ruby L. Nugent, dental medicine liaison librarian at the University of Nevada, Las Vegas, recalled when Lisa was her supervisor:

When I stepped into Lisa's office and closed her door, I was immediately at ease. I knew I could share anything I needed to and Lisa would be there to talk me through it. Lisa was not only an exceptional supervisor and mentor to me, but she is one of the most incredible humans I have ever known.

Indeed, coauthor Brittany R. Heer, health sciences librarian at Butler University, echoes this sentiment from her time at Strauss Health Sciences Library:

As I was finishing my last classes in library school, Lisa would periodically check in on me, give me words of encouragement, and be my cheerleader. She was always available to bounce ideas off of and help me prepare for interviews and presentations. Lisa's warmth, kindness, and supportive nature are qualities for which I am forever grateful. She truly defines what it means to be a leader who helps others rise to their potential.

Lisa's path in librarianship has clearly prepared her for her latest role as she steps up to the podium as the 2020–2021 MLA president: she is poised and ready to lead with openness, compassion, and professionalism. Lisa does admit that serving as MLA president at this time is a little “scary,” particularly with the difficulties faced by the cancellation of the in-person MLA '20, the MLA annual meeting, in Portland, Oregon, due to the COVID-19 pandemic this past spring. These changes have not only placed strain on the organization financially, but also have tested the capacity of MLA's administration and leadership. She is grateful for their hard work and dedication, noting along with the collective voices of the membership that MLA will be a stronger association for it.

Lisa's library career thus far has been one of challenges and successes, an ongoing adventure that she happily accepts and strives to embrace as she embarks on her MLA presidential journey. However, Lisa is quick to acknowledge that she did not do it alone. She has had many mentors and mentees, colleagues and “frolleagues,” who have supported her endeavors and celebrated her professional successes.

But there is one person that Lisa regrets not having the opportunity to thank: “I wish I knew the name of that reference librarian at the Phoenix Public Library. I wish I could tell her how grateful I am for pointing me towards librarianship.”

